# Lower Respiratory Tract Infections Associated with Rhinovirus during Infancy and Increased Risk of Wheezing during Childhood. A Cohort Study

**DOI:** 10.1371/journal.pone.0069370

**Published:** 2013-07-31

**Authors:** Cristina O’Callaghan-Gordo, Quique Bassat, Núria Díez-Padrisa, Luis Morais, Sónia Machevo, Tacilta Nhampossa, Llorenç Quintó, Pedro L. Alonso, Anna Roca

**Affiliations:** 1 Barcelona Centre for International Health Research, (CRESIB, Hospital Clínic-Universitat de Barcelona), Barcelona, Spain; 2 Centro de Investigação em Saúde da Manhiça, Manhiça, Maputo, Mozambique; 3 Medical Research Council Unit, Banjul, The Gambia; NIAID, United States of America

## Abstract

**Background and Objectives:**

Although association between respiratory syncytial virus infection and later asthma development has been established, little is known about the role of other respiratory viruses. Rhinovirus was considered a mild pathogen of the upper respiratory tract but current evidence suggests that rhinovirus is highly prevalent among children with lower respiratory tract infections (LRTI). The aim of the study was to evaluate whether LRTI hospitalization associated with rhinovirus during infancy was associated with an increased risk of wheezing – a proxy measure of asthma – during childhood.

**Methods:**

During a 12 months period, all infants <1 year admitted to Manhiça District Hospital with symptoms of LRTI who survived the LRTI episode, were enrolled in the study cohort. Nasopharyngeal aspirates were collected on admission for viral determination and study infants were classified according to presence or not of rhinovirus. The study cohort was passively followed-up at the Manhiça District Hospital for up to 4 years and 9 months to evaluate the association between LRTI associated with rhinovirus in infancy and wheezing during childhood.

**Findings and Conclusions:**

A total of 220 infants entered the cohort; 25% of them had rhinovirus detected during the LRTI episode as opposed to 75% who tested negative for rhinovirus. After adjusting for sex and age and HIV infection at recruitment, infants hospitalized with LRTI associated with rhinovirus had higher incidence of subsequent visits with wheezing within the year following hospitalization [Rate ratio=1.68, (95% confidence interval=1.02-2.75); Wald test p-value = 0.039]. No evidence of increased incidence rate of visits with wheezing was observed for the remaining follow-up period. Our data suggest a short term increased risk of wheezing after an initial episode of LRTI with RV.

## Introduction

Asthma is the most common chronic disease in children and according to the International Study of Asthma and Allergies in Childhood (ISAAC) phase three, its prevalence is increasing globally [1]. Risk factors for asthma are diverse and include, amongst others, air pollution, passive smoking and genetic factors [2,3]. Moreover, several studies have shown that lower respiratory tract infections (LRTI) during infancy also predispose to asthma later in life [4,5].

Viral respiratory infections can produce wheezing in all age groups, but particularly in children [6]. Most viral-related wheezing episodes during infancy will often disappear or cease causing bronchospasm during school years. However, in some children wheezing is the first clinical manifestation of subsequent asthma [7]. Therefore, viral infections in infancy may play an important role in development of asthma later in life. Direct association of former viral infection and asthma has been observed for respiratory syncytial virus (RSV) [8,9], the most common cause of LRTI in children [10,11], but little is known about the role of other respiratory viruses.

Until recently, rhinovirus (RV) was considered a mild pathogen of the upper respiratory tract, essentially responsible for the common cold [12]. Current evidence suggests that RV can also infect the lower respiratory tract [13] and can be highly prevalent in children with LRTI, on its own or concomitantly to other viral co-infections [14–19] in both developed and developing countries. The association between RV infection and subsequent risk of asthma has also been shown in infants hospitalized with wheezing or at increased risk of developing allergies and asthma or atopy according to parental history [20–26]

Data on RV from sub-Saharan Africa are scarce [18,19,27–29] and the association between early RV infections and subsequent asthma has not been studied in developing countries. The aim of this article is to estimate the incidence of wheezing, as a proxy measure of asthma, in a cohort of infants hospitalized with LRTI in a rural area of southern Mozambique, followed after discharge until childhood using passive case detection at the same hospital, and to evaluate if LRTI associated with RV during infancy increases risk of wheezing during childhood.

## Methods

### Study area and population

The study was conducted by the Centro de Investigaçaõ em Saúde da Manhiça (CISM) and Barcelona Centre for International Health Research (CRESIB) at the Manhiça District Hospital (MDH), the referral health facility for Manhiça District, a rural area of Maputo Province in Southern Mozambique. A complete description of the area can be found elsewhere [30,31]. Briefly, Manhiça District has an estimated population of 140.000 inhabitants. The climate of the area is subtropical with 2 distinct seasons: a warm and rainy season between November and April and a cool and dry season during the rest of the year. Malaria transmission is perennial, peaking during the rainy season [32]. In 2004, the HIV prevalence among pregnant women attending the antenatal clinic was 23.6% [33] and the estimated HIV prevalence among newborns was between 2.9 and 8% [19]. During the study period, Haemophilus influenzae b (Hib) and pneumococcal conjugated vaccine were not part of the Mozambique’s Expanded Programme of Immunization.

### Demographic surveillance in the area

A demographic surveillance system (DSS) was established in a 500 km^2^ area surrounding the centre in 1996, and during the study period followed a population of around 85.000 people. Each person living within the DSS area is issued with a unique permanent identification number. Information on vital events is collected by six-monthly household visits [30].

### Clinical surveillance and clinical management

Since 1997, MDH and CISM have jointly operated a round-the-clock morbidity surveillance of all paediatric visits at the outpatients department and of all admissions to the ward. As part of the ongoing morbidity surveillance, clinical signs and symptoms are recorded in standardized forms and determination of malaria parasites and invasive bacterial infections (IBI) are evaluated. For children living in the DSS area the unique permanent identification number is also recorded and allows linkage of incident diseases with the demographic databases.

### Study design

This analysis is part of a larger study conducted in Manhiça District Hospital (MDH) between September 2006 and September 2007. During that period, participation in the study was offered to all children <5 years old hospitalized at MDH with symptoms of LRTI, defined as cough and/or increased respiratory rate (according to WHO definition [34]) and at least one of the following: indrawing, nasal flaring, grunting or crackles detected trough auscultation. A nasopharyngeal aspirate was collected to all study children for viral determination by four different polymerase chain reactions previously described [19]. Other tests performed to all recruited children included blood culture and HIV screening. Details on the study design can be found elsewhere [19,35]. Informed written consent was obtained from each child’s mother or caregiver before study enrolment. The study was approved by the Mozambican National Bioethics Committee and the Institutional Review Board of the Hospital Clinic of Barcelona (Barcelona, Spain).

For the current analysis we retrospectively selected all children recruited for the former study who reside in the DSS area, were <1 year old at the time of hospitalization and were discharged alive after their LRTI episode. These children formed the study cohort and were passively followed from date of study enrolment until the 20^th^ of June 2011 to detect subsequent visits to MDH outpatients department with wheezing. Date of study enrolment for children with more than one LRTI hospitalization during the 12 months recruitment period was: i) date of the first LRTI hospital admission if RV was not detected in any of the admissions or ii) date of the first admission with RV detected if RV was detected in at least one of the admissions. Detection of visits with wheezing at the outpatients department was done using the morbidity surveillance system established at the MDH.

### Data management and analysis

The main exposure under study was detection of RV on the nasopharyngeal sample collected during the LRTI hospitalization. The baseline characteristics of children with and without RV detection at enrolment (exposed and non-exposed children, respectively) were compared using Chi-square tests. Odds ratio (OR) and 95% confidence intervals (95%CI) were estimated using logistic regression.

Crude incidence rates and rate ratios (IRR) of visits with wheezing in exposed and non-exposed children were calculated using a Poisson regression model. Time at risk was defined as the time from study enrolment to one of the following events: migration, death, or end of the follow-up period. After one visit with wheezing children were considered as not being at risk during an arbitrary lag period of 15 days to avoid taking into account potential multiple visits during the same episode of wheezing. Multiple events in the same child were taken into account by fitting Random Effects model to the Poisson regression model. Data was set defining origin date as date of study enrolment (see study design) and dates of entry to each risk period were defined as date of study enrolment and date of finalization of each lag period. Lexis expansion was used to split individual’s follow-up time in years since LRTI hospitalization to study the effect of time since RV detection and incidence of wheezing. A Random Effects Poisson regression model was used to calculate adjusted IRR for hospital visits with wheezing in exposed (RV detected) children versus non-exposed (no RV detected) children. The model included the following potential confounders: sex and age and HIV status at recruitment. Data were stratified by time since LRTI hospitalization.

All analyses were performed using STATA/SE 11 (Stata-Corp 2009, Stata: Release 11, Statistical Software, College Station, TX).

## Results

### Study profile

Between the 20^th^ of September 2006 to the 19^th^ of September 2007, 566 children <1 year were hospitalized in MDH with LRTI; 220 of them [39% (220/566)] resided in the DSS area and were discharged alive after their LRTI episode, and therefore were eligible to enter the cohort. Fifty-four children of the cohort [25% (54/220)] had RV detected during LRTI hospitalization and 166 children of the cohort [75% (166/220)] were RV negative. Baseline characteristics of children in the cohort according to RV detection are presented in table 1. Prevalence of HIV infection was higher among children with RV detected [OR=2.66 (95%CI=1.23-5.78); p-value = 0.038]. Fifty-nine per cent (130/220) of children were followed-up until the 20^th^ of June 2011 and the remaining children [41% (90/220)] were followed for a shorter period either because they migrated out of the DSS area [54% (49/90)] or died before this date [46% (41/90)]. Sixty-two per cent (56/90), 17% (15/90), 13% (12/90) and 8% (7/90) of loss to follow-up occurred during the first, second, third and last year respectively. Median time-at-risk per individual was 4.01 years, with no differences between exposed and non-exposed children (4.05 years and 3.61 years, respectively; p-value = 0.117). Baseline characteristic of infants who did not complete the follow-up period were compared with those who completed the follow-up period; differences were found with respect to the proportion exposed versus non-exposed [19% (25/130) vs. 32% (29/90), p-value = 0.028)[and HIV [7% (9/130) vs. 31% (31/90), p-value < 0.001)] with the prevalence of these conditions being higher among children lost to follow-up.

**Table 1:** Baseline characteristics of infants in the study cohort and crude analysis of risk factors according to RV detection.

**Variable**		**Total (n=220) n (%)**	**RV - (n=166) n (%)**	**RV + (n=54) n (%)**	OR**	**95% CI**			p-value***
**Sex**	Boy	130 (59)	93 (56)	37 (69)	1				
	Girl	90 (41)	73 (44)	17 (31)	0.58	0.30	-	1.13	0.107
**Age (months)**	≤ 3	82 (37)	67 (40)	15 (28)	1				
	4-≤6	56 (25)	41 (25)	15 (28)	1.63	0.72	-	3.69	
	7-≤12	82 (37)	58 (35)	24 (44)	1.13	0.53	-	2.42	0.243
**Season of birth**	Dry	102 (46)	79 (48)	23 (43)	1				
	Rainy	118 (54)	87 (52)	31 (57)	1.22	0.66	-	2.28	0.523
**Coinfections**									
Other viruses*	No	151 (69)	110 (66)	41 (76)	1				
	Yes	69 (31)	56 (34)	13 (24)	1.76	0.31	-	1.26	0.186
IBI	No	173 (79)	130 (78)	43 (80)	1				
	Yes	14 (6)	10 (6)	4 (7)	1.21	0.36	-	4.07	
	No data	33 (15)	26 (16)	7 (13)	0.81	0.33	-	2.01	0.849
Parasitemia	No	197 (90)	148 (89)	49 (91)	1				
	Yes	20 (9)	16 (10)	4 (7)	0.75	0.24	-	2.37	
	No data	3 (1)	2 (1)	1 (2)	1.51	0.13	-	17.13	0.837
HIV infected	No	162 (74)	129 (78)	33 (61)	1				
	Yes	37 (17)	22 (13)	15 (28)	2.66	1.23	-	5.78	
	No data	21 (9)	15 (9)	6 (11)	1.56	0.56	-	4.36	0.038
**Otherconditions**									
Malnutrition	No	121 (55)	93 (56)	28 (52)	1				
	Moderate	78 (35)	58 (35)	20 (37)	1.15	0.59	-	2.22	
	Severe	21 (10)	15 (9)	6 (11)	1.16	0.40	-	3.40	0.834
Wheezing	No	162 (74)	126 (76)	36 (67)	1				
	Yes	58 (26)	40 (24)	18 (33)	1.58	0.80	-	3.09	0.183

* Viruses included (number of viruses detected): RSV (n=24), influenza (n=7), adenovirus (n=15), human metapneumovirus (n=14), parainfluenza virus 1, 2, 3 and 4AB (n=6) and enterovirus (n=7). ** OR for age and malnutrition were calculated by comparing each category with the previous one rather than with the basal category. *** Wald test

### Crude analysis

For the 220 study children, we recorded 193 visits with wheezing during the follow-up period. Fifty-four per cent (119/220) of participants had no episode of wheezing, 25% (56/220) had one episode and 20% (45/220) had more than one episode. The estimated regression model provided strong evidence of within-child clustering of visits with wheezing (Likelihood ratio test (LRT) for clustering p-value < 0.001).

The crude incidence rate of visits with wheezing in the study cohort was 29.63/100 (95%CI=25.73-34.12) person year-at-risk (pyar). Children with RV detected on admission had a 75% higher incidence rate of visits with wheezing during the follow-up period [IRR=1.75 (95%CI=1.07-2.86); p-value = 0.026].

### Adjusted analysis

IRR of hospital visits with wheezing for RV positive children in comparison to RV negative children, adjusted for sex, age at time of hospitalization (study enrolment) and HIV infection was 1.67 (95%CI=1.02-2.73; p-value = 0.041). Adjusted IRRs stratified by time since study enrolment are presented in table 2. Results show that LRTI associated with RV during the first year of life increases the incidence rate of visits with wheezing during the year following the LRTI episode by 68% [IRR=1.68, (95%CI=1.02-2.75); p-value = 0.039]. This association seems to disappear after one year of LRTI hospitalization; no evidence of increased incidence rate of visits with wheezing was observed during the remaining follow-up period.

**Table 2:** Crude and adjusted incidence rate (IR) and incidence rate ratios (IRR) of recurrent wheezing according to RV detection during infancy by time since study enrolment (LRTI hospitalization).

**Time since study enrolment (years)**	**RV**	**n^#^**	**Num. of visits with wheezing**	pyar*	**IR (per 100 pyar)**	**Crude IRR**	**95% CI**	**p-value^§^**	**Adjusted IRR** ^±^	**95% CI**	**p-value^§^**
Overall	No	166	139	514.93	26.99	1			1		
	Yes	54	54	136.36	39.60	1.75	1.07-2.86	0.026	1.67	1.02-2.73	0.041
< 1	No	166	81	142.63	56.78	1					
	Yes	54	39	42.11	92.61	1.67	1.03-2.72	0.037	1.68	1.02-2.75	0.039
1 - < 2	No	131	32	125.98	25.40	1					
	Yes	33	11	31.33	35.11	1.51	0.70-3.25	0.291	1.53	0.71-3.32	0.278
2 - < 3	No	119	17	113.13	15.03	1					
	Yes	30	3	28.37	10.57	0.76	0.21-2.74	0.677	0.77	0.21-2.78	0.691
3 - < 5	No	109	9	133.19	6.76						
	Yes	28	1	34.55	2.89	0.46	0.06-3.77	0.471	0.47	0.06-3.84	0.481

^#^ Subjects in each category, * persons-years at risk, ^§^ Wald Test, ^±^ Adjusted for age, sex and HIV infection

## Discussion

To the best of our knowledge, this is the first study assessing the association between LRTI associated with RV in infancy and the presence of wheezing during childhood in Africa. Our data suggest a short term increased risk of wheezing after an initial episode of LRTI with RV.

Results from the current study indicate that infants hospitalized with LRTI associated with RV have more than two thirds higher risk of wheezing during the first year following hospitalization. These results are in agreement with previous studies evaluating the same association in developed countries [20–26]. RV might damage the respiratory tract of children, causing remodelling of their airways and leading to subsequent wheezing episodes and asthma. The design of the current study does not allow evaluation of the temporality of the association (children entered the cohort when they had LRTI associated with RV), and therefore we cannot discard that RV cause LRTI in infants with underlying lung impairment, who already have a tendency for wheezing. However, the entry criterion for infants in our study was hospitalization for LRTI regardless of the presence of other risk factors for asthma, and only about a quarter of them had wheezing at hospitalisation. In contrast, previous studies in developed countries were conducted among children already at increased risk of asthma, since they either had presence of wheezing during LRTI [20–22] or parental history of asthma or respiratory allergies [23–26]. Information on family risk factors for asthma was not available in our study.

After the first year of follow-up, the association between exposure and wheezing episodes was lost. The end of the association after the first period could be attributed to a real decrease of the effect of RV on subsequent wheezing when children get older, as a consequence of the maturation of their respiratory system [36]. This is supported by the decline in the number of episodes observed in both study groups after the first and second year of follow-up.

The current analysis had several challenges. Due to the observational nature of the study, the two groups of children differed in some important baseline characteristics. HIV prevalence was higher among the exposed cohort. HIV is associated with increased risk of LRTI associated with RV [19] and although we adjusted the model for HIV infection, residual confounding might remain as data on HIV status was not available for all study children. Mortality among HIV infected children was very high during the follow-up period which was translated into a higher lost to follow-up in the exposed group of children. The association under study may have been diluted by our selection of the non-exposed group as we used LRTI hospitalized infants without RV detected but we knew that some infants had LRTI associated with organisms also known to increase risk of subsequent wheezing [19] such as RSV [8,9,11,37]. The main reason for selecting hospitalized children with similar severity as the non-exposed group is that we can be certain that guardians of children from our exposed and non-exposed groups have similar heath seeking behaviour. On the contrary, selection of healthy controls as non-exposed group could have increased differences between groups as episodes were passively recorded and hospital attendance could have been lower among children recruited in the community. We did not stratify our analysis by RV specie (A, B or C). Differences in risk of wheezing according to RV specie do not seem probable as association between clinical features/disease severity and RV specie has not been detected in studies conducted in Africa ( [38], O’Callaghan-Gordo et al personal communication).

## Conclusions

Despite the challenges discussed above, results shows that in developing countries with high prevalence of HIV infection early RV infection in the context of LRTI can induce a higher risk of subsequent wheezing episodes during childhood. Therefore morbidity associated with RV goes beyond severe viral episodes during infancy as it has midterm effects. Larger studies are needed to allow more robust statistical evaluations and quantify the length of the effect. More important, the role of HIV needs to be further evaluated.
